# Identification of the Major ACE-Inhibitory Peptides Produced by Enzymatic Hydrolysis of a Protein Concentrate from Cuttlefish Wastewater

**DOI:** 10.3390/md12031390

**Published:** 2014-03-10

**Authors:** Isabel Rodríguez Amado, José Antonio Vázquez, Pilar González, Diego Esteban-Fernández, Mónica Carrera, Carmen Piñeiro

**Affiliations:** 1 Group of Recycling and Valorisation of Waste Materials (REVAL), Marine Research Institute (IIM-CSIC), r/Eduardo Cabello, 6. Vigo, Galicia 36208, Spain; E-Mails: sabelara@iim.csic.es (I.R.A.); pgonzalez@iim.csic.es (P.G.); 2 Department of Chemistry, Humboldt-Universitaet zu Berlin, Brook-Taylor Strasse 2, Berlin 12489, Germany; E-Mail: esteband@chemie.hu-berlin.de; 3 Institute of Molecular Systems Biology (IMSB), ETH Zürich, Zürich 8093, Switzerland; E-Mail: mcarrera@iim.csic.es; 4 Scientific Instrumentation Service (SICIM), Marine Research Institute (IIM-CSIC), r/ Eduardo Cabello, 6. Vigo, Galicia 36208, Spain; E-Mail: cpineiro@iim.csic.es

**Keywords:** ultrafiltration, proteolysis, ACE inhibitory peptides, cuttlefish byproducts, peptide identification, HPLC-ESI-MS

## Abstract

The aim of this work was the purification and identification of the major angiotensin converting enzyme (ACE) inhibitory peptides produced by enzymatic hydrolysis of a protein concentrate recovered from a cuttlefish industrial manufacturing effluent. This process consisted on the ultrafiltration of cuttlefish softening wastewater, with a 10 kDa cut-off membrane, followed by the hydrolysis with alcalase of the retained fraction. Alcalase produced ACE inhibitors reaching the highest activity (IC_50_ = 76.8 ± 15.2 μg mL^−1^) after 8 h of proteolysis. Sequential ultrafiltration of the 8 h hydrolysate with molecular weight cut-off (MWCO) membranes of 10 and 1 kDa resulted in the increased activity of each permeate, with a final IC_50_ value of 58.4 ± 4.6 μg mL^−1^. Permeate containing peptides lower than 1 kDa was separated by reversed-phase high performance liquid chromatography (RP-HPLC). Four fractions (A–D) with potent ACE inhibitory activity were isolated and their main peptides identified using high performance liquid chromatography coupled to an electrospray ion trap Fourier transform ion cyclotron resonance-mass spectrometer (HPLC-ESI-IT-FTICR) followed by comparison with databases and *de novo* sequencing. The amino acid sequences of the identified peptides contained at least one hydrophobic and/or a proline together with positively charged residues in at least one of the three *C*-terminal positions. The IC_50_ values of the fractions ranged from 1.92 to 8.83 μg mL^−1^, however this study fails to identify which of these peptides are ultimately responsible for the potent antihypertensive activity of these fractions.

## 1. Introduction

Cardiovascular diseases (CVD) are the main cause of death globally, accounting for approximately 17 million deaths a year, nearly one third of the total [1]. According to the World Health Organization, most cardiovascular diseases can be prevented by addressing risk factors, such as tobacco use, unhealthy diet and obesity, physical inactivity, high blood pressure, diabetes, and raised lipids [2]. Among all CVD, complications of hypertension account for 9.4 million deaths worldwide every year [3] and are the most common risk factors of heart diseases. Thus, hypertension is now considered a major health problem worldwide.

Blood pressure is regulated by the renin-angiotensin system, wherein, the angiotensin converting enzyme (ACE) hydrolyses biologically inactive angiotensin I to the potent vasoconstrictor angiotensin II, responsible for the increase in the arterial pressure. At present, the renin-angiotensin system has become a key target for drugs combating hypertension [4] and, more specifically, various synthetic ACE inhibitors are widely used to treat cardiovascular disorders. However, these synthetic drugs have been described to have some side effects [5] and, thus, natural sources of ACE inhibitors are being investigated as a milder but effective alternative for the control of high blood pressure. Among the origins of these molecules are food-derived peptides from different sources, such as cheese whey [6], gelatin [7], and different meat and fish proteins [8]. 

Marine biodiversity is a valuable source of molecules with diverse biological activities. Hence, interest has focused on the search of bioactive compounds, including ACE inhibitors, from marine sources. Peptides with ACE-inhibitory activity have been isolated from different marine species including shrimp [9], sole [10], cuttlefish [11,12], and salmon [13], among others. 

However, a more sustainable alternative providing high-value bioactive compounds is the use of fish byproducts. Among waste materials, fish processing wastewaters have been recently studied as starting materials to obtain ACE inhibitory peptides. Recently a functional concentrate from industrial shrimp (*Penaeus* spp.) cooking juice with antioxidative and antihypertensive (ACE-inhibitory) capacity has been obtained by Pérez-Santín *et al.* [14]. In addition, a screening study of different cuttlefish industrial processing wastewaters demonstrated the great potential of ultrafiltration (UF)-fractionation followed by proteolysis of cuttlefish protein concentrates as a source of peptide mixtures with antihypertensive and antioxidant activity [15]. 

The purpose of the present study was to purify and identify the major ACE inhibitory peptides produced by enzymatic hydrolysis of proteins recovered by UF from cuttlefish softening wastewater. For that purpose, the ACE inhibitory peptides from a cuttlefish byproduct hydrolysate were fractionated by sequential ultrafiltration using 10 and 1 molecular weight cut-off (MWCO) membranes. The most active fractions in the 1 kDa permeate were isolated by reversed-phase high performance liquid chromatography (RP-HPLC), their ACE-inhibitory activity quantified and the main peptides were identified by mass spectrometry.

## 2. Results and Discussion

### 2.1. Protein Recovery by Ultrafiltration-Diafiltration (UF-DF)

**Figure 1:**
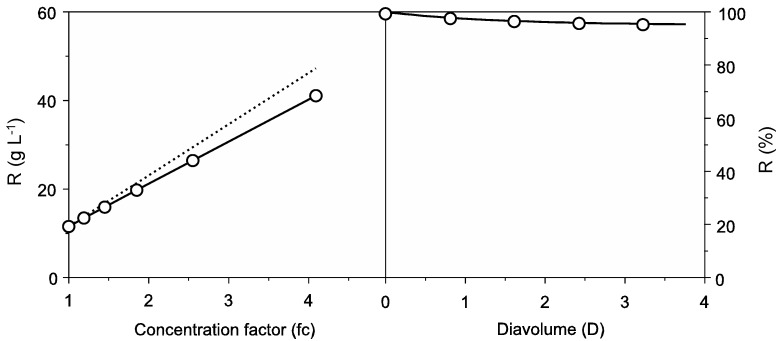
Protein recovery from cuttlefish wastewater by UF-DF at 10 kDa molecular weight cut-off (MWCO). Left: concentration of retained protein in linear relation with the volumetric concentration factor (fc) showing experimental data (points) and theoretical profiles (discontinuous line). Right: Progress of protein (○) retention with the increase of diavolume from DF process (D). For clarity, confidence intervals (in all cases less than 5% of the experimental mean value; *α* = 0.05; *n* = 2) were omitted. Equation (1) was used to fit the experimental data.

The wastewater used in this work was the result of successive cooking operations using the same water in order to obtain a high initial protein concentration. For this reason, the protein content was significantly higher than others previously reported for cuttlefish processing effluents [16,17]. Protein concentration in cuttlefish softening wastewater was 11.5 ± 0.34 g L^−1^ and reached 95.42 ± 1.22 g L^−1^ of protein content after ultrafiltration-diafiltration (UF-DF) at 10 kDa. As can be seen in Figure 1, Equation (1) was able to simulate the DF process and parameter determinations were always significant (Student’s *t* test, *α* = 0.05). Results of UF-DF using this cut-off membrane showed protein retentions higher than 95% of the initial protein content in the retentate and an overall concentration factor (fc) above 4.0. 

This result slightly improves previously published data in the treatment of cuttlefish effluents using membrane technology. An 85% reduction in the chemical oxygen demand (COD) of cuttlefish processing wastewater was observed after nanofiltration using a 550 Da cellulose acetate membrane [16]. On the other hand, a similar protein retention rate (78%) was reported in the microfiltration associated to ultrafiltration (15 kDa MWCO) of cuttlefish effluents using ceramic membranes [17]. All these studies consistently indicate a great efficiency of membrane technology for protein concentration. The use of these byproducts as a source of protein contributes to improving depuration of industrial wastewaters, reducing the treatment costs, and decreasing their contaminating effects.

### 2.2. ACE Inhibitory Activity of Cuttlefish Hydrolysate and Their UF Fractions

To obtain the ACE inhibitors, the cuttlefish protein concentrate was hydrolysed using alcalase according to optimal conditions previously reported for this substrate [12]. The extent of protein degradation by the proteolytic enzyme was estimated by assessing the degree of hydrolysis (DH) (Equation (2)). According to this calculation a high DH was observed from the beginning of the digestion, reaching a final value of 37.8% ± 0.4% after 8 h of proteolysis (Table 1). 

**Table 1:** Degrees of hydrolysis (DH) after 0.5, 2, and 8 h digestion with alcalase of a cuttlefish wastewater protein concentrate. Angiotensin converting enzyme (ACE) inhibitory activity (I_ACE_ (%)) and IC_50_ values (μg mL^−1^) of these hydrolysates, as well as retentates obtained by 10 and 1 kDa membrane and permeate of 1 kDa from the 8 h hydrolysate. I_ACE_ (%) was calculated by Equation (3). Mean values and standard deviations from triplicate samples are shown.

Values of DH, I_ACE_ (%) and IC_50_ for Hydrolysis and UF Processes				
		DH (%)	I_ACE_ (%)	IC_50_ (μg mL^−1^)
		–	37.8 ± 0.4	292.5 ± 20.3
**Hydrolysis**	0.5 h	17.5 ± 0.2	76.2 ± 3.2	214.4 ± 27.3
	2 h	23.8 ± 1.8	80.4 ± 2.3	122.5 ± 13.1
	8 h	36.4 ± 0.9	92.1 ± 0.6	76.8 ± 15.2
**Ultrafiltration**	10 kDa retentate	–	83.3 ± 0.6	273.9 ± 30.1
	1 kDa retentate	–	94.6 ± 2.9	235.6 ± 20.2
	1 kDa permeate	–	68.0 ± 1.4	58.4 ± 4.6

Indeed, the highest levels of hydrolysis were greater than those obtained from cuttlefish muscle [18] and other seafood species [13] using this enzyme. As reported before [12], this difference might be attributed to the thermal treatment of proteins recovered by UF, which tends to increase the susceptibility of proteins to enzymatic hydrolysis due to their partial denaturation. 

ACE inhibitory activity improved with increasing time of hydrolysis, reaching maximal values (92.1% ± 0.6%) after 8 h of proteolysis (Table 1). In addition, a direct relationship between ACE inhibitory activity and DH was observed in a study evaluating the ACE inhibitory activities of cuttlefish muscle protein hydrolysates prepared by treatment with various bacterial proteases [18]. In fact, the authors obtained a lower inhibitory activity (51.5% ± 1.5%) from a cuttlefish protein hydrolysate prepared using alcalase with a DH of 12.5%, *i.e.*, three-fold lower than the DH reported in the present study. However, it must be noted that hydrolysis conditions and calculation methods for the DH and ACE inhibitory varied between both studies, making difficult the direct comparison of the results. 

**Figure 2:**
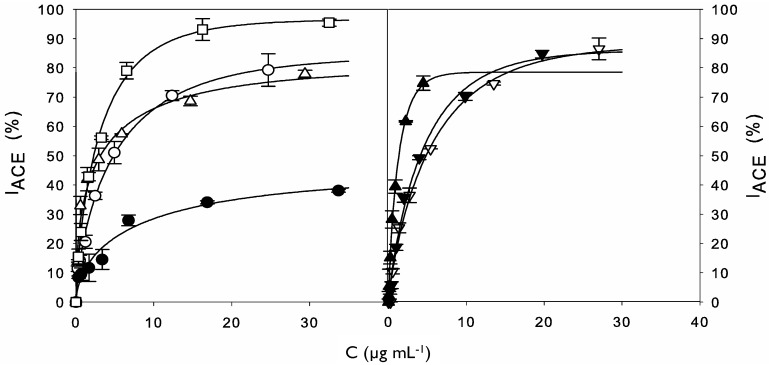
Left: Experimental data (symbols) for ACE-inhibitory activity of cuttlefish softening wastewater protein concentrate before (●) and after hydrolysis with alcalase for 0.5 (○), 2 (△), and 8 h (□). Right: Experimental data (symbols) for ACE-inhibitory activity of 10 kDa (▽) and 1 kDa (▼) retentates and 1 kDa permeate (▲) from the 8 h hydrolysate. The dose-response curves (lines) were obtained by modeling experimental data to the Equation (4).

To calculate IC_50_ values, dose-response curves (Figure 2) were plotted and accurately fitted by Equation (4), with coefficients of determination greater than 0.98.

Equation (4) allowed the direct determination of IC_50_ values using mathematical nonlinear fitting. The estimation of IC_50_ values using this model is more precise than direct interpolation or linear regression and allows the objective comparison between samples. This equation was consistent (Fisher’s *F* test; *p* < 0.05) and parameter estimations were always significant (Student’s *t* test, *α* = 0.05) (data not shown). According to this approach, IC_50_ decreased with increasing time of hydrolysis reaching the lowest value (maximal ACE inhibitory activity) after 8 h of hydrolysis. These differences revealed that the use of alcalase was efficient in releasing peptides with ACE inhibitory activity from cuttlefish protein concentrate. In fact, this endoprotease has been widely employed for the production of potent marine derived ACE inhibitors from sources as diverse as the algae *Chlorella* *ellipsoidea* [19], salmon byproducts [20], seaweed pipefish muscle protein [21], the sea cucumber *Acaudina molpadioidea* [22], and lizard fish [23]. This enzyme is also less expensive than others commercially available, thus, being a good candidate for the industrial production of bioactive hydrolysates from marine byproducts. Values of IC_50 _for ACE inhibitory activity of whole cuttlefish hydrolysates have being previously reported for various digestive proteases [11], including alcalase [18]. These values ranged from 1.19 to 2.31 mg mL^−1^, however the direct comparison with IC_50_ values in the present study (Table 1) is not possible due to differences in the hydrolysis conditions but, also, in the methodology employed for ACE inhibitory activity determination and calculation.

**Figure 3:**
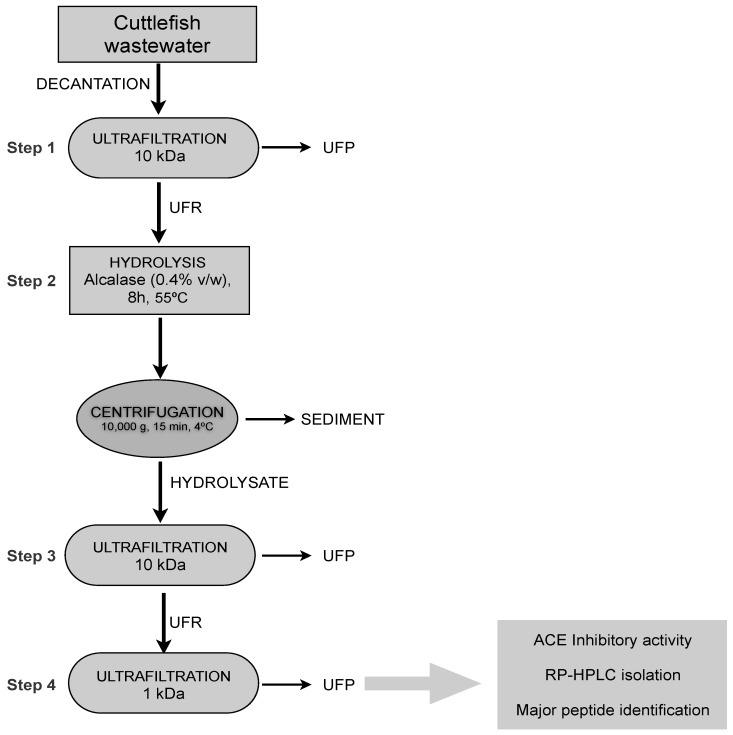
Process of ACE inhibitory peptide production from a protein enriched fraction recovered from cuttlefish wastewater by UF-DF at 10 kDa (Step 1) followed by hydrolysis with alcalase (0.4% v/v) for 8 h at 55 °C (Step 2). Then, the hydrolysate was fractionated using a sequentially UF through membranes of 10 (Step 3) and 1 kDa (Step 4). UFP: Permeates of UF; UFR: retentates of UF.

The 8 h hydrolysate, which led to the highest ACE inhibitory activity (lowest IC_50_), was fractionated in an attempt to isolate the active peptides. The use of UF has proven to be a valuable resource for hydrolysate fractionation, significantly decreasing the IC_50_ value of peptide mixtures obtained from different sources [6,24,25,26]. For this reason, the 8 h hydrolysate was sequentially ultrafiltered through membranes at 10 and 1 kDa MWCO (Figure 3). Then, permeate and retentates ACE inhibitory activity was determined. As summarized in Table 1, the lowest IC_50_ value was associated to 1 kDa permeate (58.4 ± 4.6 μg mL^−1^), indicating that the maximum contribution to ACE-inhibitory activity is due to the peptides with MW below 1 kDa. Similarly, the ACE inhibitory activity was significantly higher in the lowermost MW fraction (<1 kDa) from collagenous residues from squid skins hydrolyzed with esperase [25].

This observation is in good agreement with previous studies reporting greater *in vitro* activity of low than high molecular weight peptides from different protein origin [13,18]. 

### 2.3. Identification of Major ACE Inhibitory Peptides

**Figure 4:**
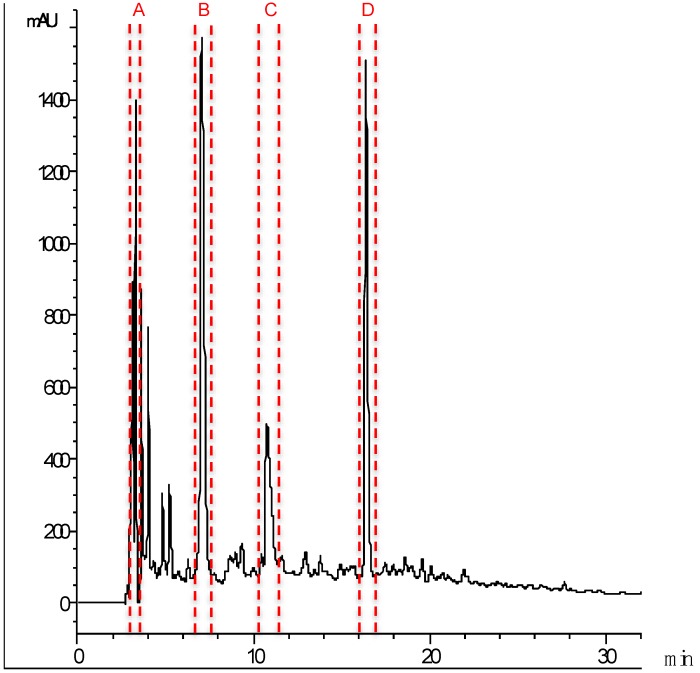
Peptide profile of the 8 h hydrolysate that was permeated through the 1 kDa ultrafiltration membrane after RP-HPLC separation. Lines indicate the peaks corresponding to the four active fractions (A–D).

The ACE inhibitory peptides in the 1 kDa permeate from cuttlefish hydrolysate were separated by RP-HPLC on a C18 column. The chromatogram (Figure 4) was divided into sixty-five fractions (2 mL) that were freeze-dried and their ACE inhibitory activity determined after resuspension in milliQ water and pH adjustment to 7.0. Four fractions showing the highest activity were collected separately under optimized separation conditions that allowed a better resolution of the peaks, *i.e.*, lower flow rate and a narrower acetonitrile gradient. The separation procedure was successively repeated to obtain a sufficient protein concentration for further analysis. The most active fractions (Figure 4A–D) were freeze-dried and their ACE inhibitory activity and IC_50_ values determined (Table 2).

The chemical characterization of each RP-HPLC fraction was carried out using mass spectrometry, in order to identify the main peptides contributing to the ACE inhibitory activity measured in these samples. According to these results (Table 2), among the four fractions only two (C and D) matched the protein databases and the other two fractions (A and B) had to be sequenced *de novo*. 

**Table 2:** Major identified peptides corresponding to the four more active fractions after semi-preparative fractionation by reversed-phase high performance liquid chromatography (RP-HPLC) of the 1 kDa ultrafiltration permeate from the 8 h hydrolysate. The IC_50 _(μg mL^−1^) value and the protein source of these fractions are shown.

Fraction	Peptides	Protein Source	Mass (Da)	IC_50 _(μg mL^−1^)
A	GNALVFLLP		943.57	1.92 ± 0.40
	FALASSLVN		921.53	
	SPSIAPAL		755.40	
	GLFAAHRK		899.54	
	FTPESLAI		877.50	
	DAAIKTLTK		960.60	
	MMQLRKA		877.50	
	RALKIPAM		899.54	
	FTVKPVSR		933.59	
	SSSKAKKKP		960.60	
	KAVPIAIL		824.58	
	TRAASCP		705.30	
	INKAVTGLK		943.57	
	PEGSIRP		755.40	
B	VEDAEVGKK		974.52	4.00 ± 0.19
	FAGDDAPRA		919.43	
	AGSVNKSK		790.44	
	KAGSELGL		774.44	
	QSLEVSK		790.43	
	KEAEVSK		790.43	
	GAEVTVSK		790.43	
	QEVSLSK		790.43	
	AGEVSLSK		790.43	
	NFGCSVK		754.35	
	SVCGGFGK		754.35	
	AGDDPAR		701.32	
C	ADQSEGALQK	Myosin HC	1045.5	8.63 ± 0.86
	ISEQEASQR	Tropmyosin	1046.5	
	LGEGGRSTHE	Myosin HC	1041.5	
	RLKEAENR	Actin	981.5	
	TDQLGEGGRS	Myosin HC	1018.5	
D	DEDATGVIR	Tn C	974.47	7.18 ± 2.44
	GTDPEDALRN	Myosin RLC	1086.49	
	GVNLEDAKRS	Myosin HC	1087.56	
	LTEAPLNPK	Actin	981.55	
	REDIDGNIK	Myosin LCK	1058.54	

The peptides identified in the fractions C and D are derived from muscular protein fragments of the cuttlefish mantle. Softening of fish muscle consists on the heating and addition of salts to solubilize myofibrillar proteins, improving the water-fat-retention and leading to acceptable rigidity/elasticity of the muscle gels [27]. Thus, as expected and since the cuttlefish hydrolysate was prepared from a protein concentrate recovered from softening wastewater, myofibrillar proteins, such as troponin, actin, and myosin were identified as protein sources from the identified peptides (Table 2). Antihypertensive peptides isolated from other squid proteins hydrolysates, such as collagen [27] and gelatin [7,28], have also been reported. These studies identified peptides sequences of the major ACE inhibitory fractions with a high content in proline, glycine, and leucine residues and suggesting the presence of this last amino acid could play an important role in the ACE-inhibitory activity [28].

As is known, peptide sequence greatly contributes to its biological activity. The structure–function relationship for ACE inhibitory peptides is not fully characterized, although recently published papers deal with the structure-activity relationships of di and tripeptides [29,30]. Some of the information available about this topic indicates that the three amino acids in the *C*-terminal peptide sequence are particularly important for their binding to ACE, as they might interact with the active site of the enzyme [31]. For instance, this enzyme prefers hydrophobic amino acids, including aromatic residues (phenylalanine, tryptophan, and tyrosine) and branched chain amino acids (valine, leucine, and isoleucine) in one of the three positions of the *C*-terminal [32]. In this study, all of the peptides identified in the four potent ACE inhibitory fractions contained at least one hydrophobic residue, being the most abundant branched chain amino acids (Table 2). 

On the other hand, ACE has low affinity for peptides with dicarboxylic amino acids in this position or those containing the amino acid proline in the penultimate position [33], although proline in final or penultimate position of the *C*-terminal peptide sequence promotes enzyme binding [34]. In fact, several identified ACE inhibitory peptides had a proline residue in one of these positions of *C*-terminal, although this is neither sufficient nor essential to confer activity [18]. Furthermore, the positively charged amino acids, such as arginine and lysine at the *C*-terminal sequences were also reported to contribute towards ACE inhibitory or antihypertensive effect [35]. As can be seen in Table 2, among the identified peptides many of them contained one of these residues in the *C*-terminal. This was especially evident in fraction B, where ten of the twelve peptides identified contained lysine or arginine in last position. In agreement with other published papers, these findings confirm that peptide sequences are greatly important to define their ACE inhibitory activity (IC_50_ values). However, the differences in the composition of the peptides identified in this study fail to explain the differences found in the ACE inhibitory activity and, thus, further studies are needed to define which of these peptides are ultimately responsible for the potent antihypertensive activity of these fractions.

## 3. Experimental Section

### 3.1. Cuttlefish Processing Wastewater

Wastewater from the softening treatment during industrial manufacturing of cuttlefish was kindly provided by Frinova S.A., (Porriño, Galicia, Spain). The average content of suspended solids of this water was ranged among 0.3–0.7 g L^−1^ and the solid residue was around 10–15 g L^−1^. The water was decanted to discard the particulate matter, sampled for analytical determinations, and stored at −20 °C until further use. 

### 3.2. Total Protein Analysis

Protein concentration was determined by the bicinchoninic acid assay (BCA, Pierce, Rockford, IL, USA) using bovine serum albumin as standard. Briefly, 200 μL of the BCA working reagent were mixed with 25 μL of sample. The mixture was incubated for 30 min at 37 °C and the absorbance taken at 562 nm using a Multiskan Spectrum Microplate Spectrophotometer (Thermo Scientific, Waltham, MA, USA).

### 3.3. Ultrafiltration-Diafiltration Process

Cleaned cuttlefish wastewater was subjected to ultrafiltration-diafiltration using a Prep/Scale-TFF polyethersulfone cartridge (Millipore Corporation, Bedford, MA, USA) of 10 kDa molecular weight cut-off (MWCO). The operation mode was the following: an initial phase of ultrafiltration (UF) at 40 °C with total recirculation of retentate was performed, immediately followed by a diafiltration (DF) process. During UF, the inlet pressure remained constant to determine the flow rate drops due to the increased concentration of protein in the retentate and to possible membrane fouling. The final retentate (after DF) was frozen at −20 °C for further hydrolysis and permeate from the DF phase was discarded.

For the modeling of the membrane process, we assumed that in the DF with constant volume (filtration flow = water intake flow), the concentration (or the total amount) of a permeable solute in the retentate followed first order kinetics [15]:

*R* = *R_f_* + *R*_0_ exp[−(1 − *s*) *D*] (1)

where, *R* is the concentration of permeable protein in the retentate (% from the level at initial DF), *R_0_* the initial concentration (%), *R_f_* is the final and asymptotic concentration (%), *D* is the relative diavolume (volume of added water/constant retentate volume) and *s* is the specific retention of protein with variation between 0 (the solute is filtered as the solvent) and 1 (the solute is totally retained). Thus, using normalized values (%): *R_0_* + *R_f_* = 100, being *R_0_* = 0 if all protein is permeable. 

### 3.4. Preparation of Enzymatic Hydrolysates

The cuttlefish wastewater concentrate (retentate) was pre-incubated at 55 °C for 10 min prior to enzymatic hydrolysis using Alcalase 2.4 L from Novo Co. (Novozyme Nordisk, Bagsvaerd, Denmark). The enzyme to substrate ratio (E/S) was 0.4% (v/w) in relation to retentate weight and the reaction was performed in a temperature-controlled water bath under constant magnetic stirring [12]. Samples were taken after 0.5, 2, and 8 h of hydrolysis, boiled for 15 min to inactivate the protease and then cooled in an ice-water bath for at least 30 min. Hydrolysates were centrifuged at 10,000× *g* for 15 min (4 °C) in an Avanti J-26 XP centrifuge (Beckman Coulter, Inc., Miami, FL, USA) and supernatants were stored at −20 °C until further analysis of their degree of hydrolysis and antihypertensive activity.

### 3.5. Degree of Hydrolysis Determination

The degree of hydrolysis (DH) was defined as the ratio of the released tyrosine to the initial tyrosine in the retentate [12], according to the following expression:

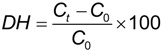
(2)
where, *C_t_* is the tyrosine concentration (g L^−1^) of the hydrolysate at time *t* and *C_0_* is the tyrosine concentration (g L^−1^) of the sample at time zero. Tyrosine was measured by the method described in Barker and Worgan [36]. 

### 3.6. Angiotensin I-Converting Enzyme (ACE) Inhibition Assay

The antihypertensive activity was determined using a modified version of the method described by Shalaby *et al.* [37] with *N*-[3-(2-Furyl) acryloyl]-l-phenylalanyl-glycyl-glycine (FAPGG) as substrate. Briefly, 10 μL of sample were mixed with 10 μL of ACE solution (0.5 U mL^−1^) in each well of a 96-well microtiter plate. The reaction was started by adding 150 μL of substrate (0.88 mM FAPGG in 50 mM Tris-HCl, pH 7.5, 0.3 M NaCl) pre-heated at 37 °C. The absorbance at 340 nm (A340) was acquired at time intervals of 30 s for 30 min in a Multiskan Spectrum Microplate Spectrophotometer (Thermo Scientific, Waltham, MA, USA). The control consisted of samples containing 10 μL of buffer (50 mM Tris-HCl, pH 7.5, 0.3 M NaCl) instead of protein hydrolysate. Samples and controls were performed in triplicate.

ACE-inhibitory capacity (I_ACE_) was calculated as a function of the average slope of decrease in absorbance with time and expressed as percent of enzyme inhibition according to the following expression:

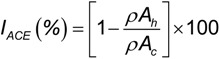
(3)
where, *I_ACE_* is the ACE-inhibitory capacity (%), *ρA_h_* is the slope of decrease in A340 in the presence of inhibitor (hydrolysate) and *ρA_c_* is the slope of decrease in A340 in the absence of inhibitor (control). 

For the calculation of the protein concentration causing a 50% ACE inhibition (IC_50_), dose-response curves were obtained assaying different concentrations of hydrolysates. IC_50_ values were calculated by fitting the dose-response curves of I_ACE_ *vs.* protein concentration to a mechanistic model developed by Estévez *et al.* [6]:

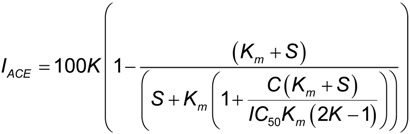
(4)
where, *K* is the maximum *I_ACE_* (%), *C* is the protein concentration (μg mL^−1^) and *IC_50_* is the concentration for semi-maximum response (μg mL^−1^). *K_m_* is the Michaelis–Menten constant of FAPGG and *S* is the concentration of this substrate in the assay, respectively. Both *K_m_* and *S* are constants under these experimental conditions, with values of 182 μg mL^−1^ and 310 μg mL^−1^, respectively [6].

### 3.7. Isolation of Major ACE-Inhibitory Peptides

The 8-h-hydrolysate was further ultrafiltered to remove peptides with high molecular weight. UF were carried out in a stirred UF cell module (Millipore Corporation, Bedford, MA, USA) under a nitrogen flow pressure of 40 psi at room temperature using separately 10 and 1 kDa MWCO membranes (Millipore Corporation, Bedford, MA, USA). Firstly, the hydrolysate was ultrafiltrate using a 10 kDa MWCO membrane followed by further fractionation of the 10 kDa permeate through a 1 kDa MWCO membrane. Samples from 10 and 1 kDa retentates and 1 kDa permeate were collected and protein concentration and ACE-inhibitory activity (IC_50_) were determined.

The 1 kDa permeate was separated by RP-HPLC on an Agilent 1200 series system (Agilent, Waldbronn, Germany) using an ACE 5 C18 column (10 × 250 mm, 5 μm). Peptides were eluted and monitored at 220 nm using water containing 0.1% trifluoric acetic acid (TFA) (mobile phase A) and acetonitrile with 0.1% TFA (mobile phase B), with a linear gradient of acetonitrile (0%–50% v/v) during 50 min. The ACE inhibitory activity was determined in 2 mL fractions collected at a flow rate of 2.5 mL min^−1^. Those fractions showing the highest ACE inhibitory activity were collected using an Agilent Zorbax Eclipse C18 column (4.6 × 250 mm, 5 μm) with a linear gradient of acetonitrile (5%–30% v/v) at a flow rate of 0.75 mL min^−1^ during 30 min. According to the chromatographic peptide profile obtained, four fractions from ten samples were defined and collected manually, frozen at −80 °C, and lyophilized for further protein quantitation, antihypertensive activity determination, and mass spectrometry identification analysis.

### 3.8. Mass Spectrometry Analysis of ACE-Inhibitory Peptides

The freeze-dried fractions were diluted in 1 mL of 1% ACN, 0.1% formic acid (FA) and 2 μL aliquots were analyzed using a C18 column coupled to an electrospray ion trap Fourier transform ion cyclotron resonance-mass spectrometer (ESI-IT-FTICR, Thermo Fisher Scientific, Bremen, Germany). A data dependent fragmentation method consisting of cycles of high resolution full scan spectra followed by ion trap fragmentation and detection of the five most intense masses was used for identification purposes. The chromatographic system (Agilent 1200 series, Waldbronn, Germany) was equipped with an Agilent SB C-18 column (0.5 × 150 mm, 5 μm). A very slow elution program, namely, 0/5, 6/5, 7/10, 32/40, 34/90 (min/%B, being mobile phase A 5% ACN, 0.1% FA and mobile phase B pure ACN, 0.1% FA), at a flow of 20 μL min^−1^, was optimized to enhance the separation of the low hydrophobic species fractionated in the previous semi-preparative chromatographic dimension.

### 3.9. Mass Spectrometry Data Processing

MS/MS spectra were searched using SEQUEST-SORCERER™ 2 package (Sage-N Research Inc., Milpitas, CA, USA), against the UniProt/SwissProt database (release 2012_10; 455.545 entries), which also included their respective decoy sequences. The following constraints were used for the searches: semi-tryptic cleavage with up to two missed cleavage sites and tolerances 1.0 Da for precursor ions and 0.5 Da for MS/MS fragments ions. The variable modifications allowed were methionine oxidation (m), carbamidomethylation of Cys and acetylation of the *N*-terminus of the protein (*N*-Acyl). The database search results were subjected to statistical analysis with the PeptideProphet algorithm (v.4.4) [38]. The FDR was kept below 1%. 

*De novo* sequencing was performed by manual interpretation of the ion series of the spectra with aid of the software packages: DeNovoX (Thermo Scientific, Waltham, MA, USA) and PEAKS Studio 6.0 (Bioinformatics Solutions Inc., Waterloo, Ontario, Canada). The parameters used for both programs were as follows: selection of the peptide charge (1+, 2+, 3+), tolerances of 0.3–0.5 Da for precursor and fragments ions, use or not of trypsin or Glu-C as proteases and three variable modifications: Methionine oxidation (m), carbamidomethylation of Cys and acetylation of the *N*-terminus of the protein (*N*-Acyl).

## 4. Conclusions

In this study, ACE inhibitory peptides were produced from cuttlefish byproduct protein hydrolysate using a combination of membrane technology (UF-DF) and enzyme proteolysis using alcalase. The sequential ultrafiltration with two membranes cut-offs (10 and 1 kDa) led to recover a remarkable antihypertensive activity. The most bioactive peptides were subsequently separated by means of RP-HPLC and characterized by HPLC-ESI-IT-FTICR. The procedure developed in the present work demonstrated to be a feasible biotechnological approach to produce antihypertensive peptides from marine wastes and to help in the depuration and valorization of wastewaters generated by marine food processing. Nevertheless, further studies (peptide synthesis, re-chromatography of the HPLC fractions, *etc.*) are needed in order to identify which among these peptide mixtures are responsible for the observed activity.
